# The Transdiagnostic Intervention for Sleep and Circadian Dysfunction (TranS-C) for serious mental illness in community mental health part 1: study protocol for a hybrid type 2 effectiveness-implementation cluster-randomized trial

**DOI:** 10.1186/s13063-023-07148-9

**Published:** 2023-03-17

**Authors:** Laurel D. Sarfan, Emma R. Agnew, Marlen Diaz, Lu Dong, Krista Fisher, Julia M. Spencer, Shayna A. Howlett, Rafael Esteva Hache, Catherine A. Callaway, Amy M. Kilbourne, Daniel J. Buysse, Allison G. Harvey

**Affiliations:** 1 Department of Psychology, University of California, Berkeley, CA Berkeley, USA; 2 RAND Corporation, Santa Monica, CA USA; 3 University of Michigan and VA Ann Arbor Healthcare System, Ann Arbor, MI USA; 4 Department of Psychiatry, University of Pittsburgh, Pittsburgh, PA USA

**Keywords:** Transdiagnostic, Sleep, Circadian, Serious mental illness, Implementation, Adaptation, Community mental health

## Abstract

**Background:**

Serious mental illness (SMI) can have devastating consequences. Unfortunately, many patients with SMI do not receive evidence-based psychological treatment (EBPTs) in routine practice settings. One barrier is poor “fit” between EBPTs and contexts in which they are implemented. The present study will evaluate implementation and effectiveness outcomes of the Transdiagnostic Intervention for Sleep and Circadian Dysfunction (TranS-C) implemented in community mental health centers (CMHCs). TranS-C was designed to target a range of SMI diagnoses by addressing a probable mechanism and predictor of SMI: sleep and circadian problems. We will investigate whether adapting TranS-C to fit CMHC contexts improves providers’ perceptions of fit and patient outcomes.

**Methods:**

TranS-C will be implemented in at least ten counties in California, USA (*N* = 96 providers; *N* = 576 clients), via facilitation. CMHC sites are cluster-randomized by county to Adapted TranS-C or Standard TranS-C. Within each county, patients are randomized to immediate TranS-C or usual care followed by delayed treatment with TranS-C (UC-DT). Aim 1 will compare TranS-C (combined Adapted and Standard) with UC-DT on improvements in sleep and circadian problems, functional impairment, and psychiatric symptoms. Sleep and circadian problems will also be tested as a mediator between treatment condition (combined TranS-C versus UC-DT) and functional impairment/psychiatric symptoms. Aim 2 will evaluate whether Adapted TranS-C is superior to Standard TranS-C with respect to provider perceptions of fit. Aim 3 will evaluate whether the relation between TranS-C treatment condition (Adapted versus Standard) and patient outcomes is mediated by better provider perceptions of fit in the Adapted condition. Exploratory analyses will (1) compare Adapted versus Standard TranS-C on patient perceptions of credibility/improvement and select PhenX Toolkit outcomes and (2) evaluate possible moderators.

**Discussion:**

This trial has the potential to (a) expand support for TranS-C, a promising transdiagnostic treatment delivered to patients with SMI in CMHCs; (b) take steps toward addressing challenges faced by providers in delivering EBPTs (i.e., high caseloads, complex patients, poor fit); and (c) advance evidence on causal strategies (i.e., adapting treatments to fit context) in implementation science.

**Trial registration:**

Clinicaltrials.gov NCT04154631. Registered on 6 November 2019. https://clinicaltrials.gov/ct2/show/NCT04154631

**Supplementary Information:**

The online version contains supplementary material available at 10.1186/s13063-023-07148-9.

## Introduction

Serious mental illness (SMI) can have devastating psychosocial and health consequences [1–3]. SMI can be operationalized based on Public Law 102–321, the National Institute of Mental Health, and prior research as the presence of at least one psychiatric disorder that leads to substantial interference with one or more major life activities [4, 5]. Unfortunately, patients with SMI treated in routine practice settings too often do not receive evidence-based psychological treatment (EBPT) [6–8]. Indeed, although there has been a proliferation in EBPTs, only a small fraction of EBPT research is translated into routine practice settings [9, 10].

Thus, large-scale implementation of EBPTs could have a tremendous positive impact. However, barriers have been identified. One barrier is that the context for implementation (e.g., routine practice settings) typically differs from the context in which EBPTs are developed (e.g., academic institutions). This causes poor “fit,” operationalized herein as provider perceptions of EBPT acceptability, appropriateness, and feasibility within their routine practice setting [11–14]. Importantly, fit predicts a host of implementation outcomes, including reach, treatment fidelity, and sustained use of treatments [15–17].

In the present study, we sought to determine if fit of an EBPT for SMI could be improved in the context of community mental health centers (CMHCs). CMHCs are major, publicly funded providers of treatment for SMI. CMHC providers carry heavy caseloads with high rates of comorbidity and complexity [18, 19]. Although it is not uncommon for CMHC providers to receive training in EBPTs, poor fit between EBPTs and CMHC contexts has contributed to infrequent use or even discontinuation [16, 20–22].

A related challenge for CMHC providers and SMI treatment is that, in the proliferation of EBPTs, many focus on treating a single diagnosis [23]. Consequently, CMHC providers—and indeed providers in most routine practice settings—would need to learn and use several different treatments to address the various diagnoses of their patients. Given high caseloads and provider perceptions that EBPTs are more time-consuming to learn and use than treatment as usual [21], these single-diagnosis treatments may reflect a significant barrier to implementing EBPTs.

A promising alternative to single-diagnosis treatments is the *transdiagnostic* approach to treatment. The transdiagnostic approach holds that disorders co-occur in part due to common processes that drive symptoms [24–26]. By targeting these common processes, transdiagnostic treatments can address the causes and symptoms of multiple disorders with a single protocol. In other words, transdiagnostic treatments may represent a path by which CMHC providers could use a single EBPT to help patients with a range of mental health problems.

Circling back to SMI treatment in CMHCs, a question arises: what common mechanisms could be targeted by a transdiagnostic treatment to address a range of SMI diagnoses? Four lines of evidence highlight *sleep and circadian problems* as an important transdiagnostic contributor to SMI. First, sleep and circadian problems, including insomnia, hypersomnia, and evening circadian preference, predate and predict a range of mental illness, including depression, substance use, anxiety, and psychosis [27–32]. Second, common cognitive (e.g., worry, rumination), behavioral (e.g., avoidance), and biological (e.g., dopaminergic and serotonergic) processes may predict and maintain both SMI and sleep and circadian problems [26, 33, 34]. Third, treatments that focus on sleep and circadian problems are concurrently associated with improvements in mental health symptoms [35–37]. Fourth, there may be substantial heterogeneity in comorbidities between SMI and sleep and circadian problems in routine practice settings. For instance, in a CMHC sample, participants with schizophrenia spectrum disorder (*n* = 50), bipolar disorder (*n* = 35), and major depressive disorder (*n* = 26) exhibited 25, 24, and 21 distinct patterns of sleep and circadian problems, respectively [19]. Moreover, over 85% of the sample met the criteria for at least one comorbidity between sleep and circadian problems, and over 80% met the criteria for one or more sleep or circadian problems at the subdiagnostic level. Of note, *number* of sleep or circadian problems—including those at the subdiagnostic level—was more predictive of impairment than *diagnostic threshold* of those problems [38]. Collectively, these lines of research support the potential value of multi-problem, transdiagnostic interventions [39].

Building on this evidence, the Transdiagnostic Intervention for Sleep and Circadian Dysfunction (TranS-C) was developed [40]. Based on the Sleep Health Framework [41], TranS-C is transdiagnostic in two ways: it targets a range of sleep and circadian problems for individuals with a range of SMI diagnoses. TranS-C is a psychosocial, skills-based, and modular approach, consisting of (a) four core modules that form the basic building blocks of sleep health, (b) four cross-cutting interventions used in every session (e.g., motivational enhancement), and (c) seven optional modules that can be integrated based on case conceptualization, patient goals, and clinical judgment (see the “Method” section for details).

The preliminary evidence for TranS-C in CMHCs has been promising. Specifically, among adult CMHC patients, TranS-C relative to usual care followed by delayed treatment with TranS-C (UC-DT) was associated with improvements in sleep and circadian problems, functional impairment, and psychiatric symptoms [35]. However, the providers delivering TranS-C were employed, trained, and supervised within an academic setting. The National Institute of Health’s stage model holds that a critical next step would be to test TranS-C in a community setting with *CMHC providers* [42]*.* This is particularly important because CMHC providers are responsible for the day-to-day delivery of clinical services, including EBPTs. Thus, ensuring that TranS-C is acceptable to CMHC providers, as well as effective for their patients, is essential. There is already promising evidence that cognitive behavior therapy for insomnia can be delivered in routine practice [43], including by non-specialists [44, 45], and the present study will build on these findings.

Pertinent to this next step, TranS-C as it is typically delivered—henceforth “Standard TranS-C”—involves relatively high-intensity procedures similar to other EBPTs. Specifically, TranS-C consists of eight weekly, 50-min sessions. Indeed, in a process evaluation, CMHC staff identified dose and complexity as barriers to implementing Standard TranS-C [46]. In response, guided by the Replicating Effective Programs framework [47], our team developed a modified version of TranS-C—henceforth “Adapted TranS-C”—to improve fit with the CMHC context (see the “Method” section for systematic development of Adapted TranS-C).

## Aims

Together, the goal of the present hybrid type 2 effectiveness-implementation study [48, 49] is to evaluate the implementation and effectiveness outcomes of TranS-C in CMHCs of counties across California in the USA. The study will be conducted in three phases. This protocol will focus on phase 1, the Implementation Phase, during which TranS-C is implemented in CMHCs via facilitation (see the “Method” section). Phase 2 is the Train-the-Trainer Phase, during which CMHC providers learn to train and supervise their peers in the delivery of TranS-C. Phase 3 is the Sustainment Phase, during which we will assess the extent to which TranS-C activities are sustained after facilitation has ceased.

During the Implementation Phase, sites are cluster-randomized by county to Adapted TranS-C or Standard TranS-C with 1:1 allocation. Then, within each county, patients are randomized to immediate TranS-C or UC-DT. The first aim is to assess the effectiveness of TranS-C, compared to UC-DT. We hypothesize that, compared to UC-DT, TranS-C (combined Adapted and Standard) will be associated with larger reductions in the primary patient outcome of sleep disturbance and the secondary patient outcomes of sleep-related impairment, functional impairment, and psychiatric symptoms. We also hypothesize that TranS-C’s benefits for functional impairment and psychiatric symptoms will be mediated by improvements in sleep and circadian problems. The second aim is to evaluate whether TranS-C treatment condition (Adapted versus Standard TranS-C) is associated with fit to the CMHC context, operationalized as provider ratings of acceptability, appropriateness, and feasibility. We hypothesize that Adapted TranS-C will be superior to Standard TranS-C with respect to the primary provider outcome of acceptability and the secondary provider outcomes of appropriateness and feasibility. The third aim is to evaluate whether better fit mediates the relation between TranS-C treatment condition and patient outcome. We hypothesize that relative to Standard TranS-C, Adapted TranS-C will be associated with greater reductions in the primary and secondary patient outcomes indirectly through higher provider ratings of acceptability, appropriateness, and feasibility. Exploratory analyses will (1) compare Adapted and Standard TranS-C on patient perceptions of credibility/improvement and select PhenX Toolkit outcomes and (2) determine whether treatment effects are moderated by risk factors including age, sex, and sleep and circadian and psychiatric symptoms at baseline [50, 51].

## Method

**Fig. 1:**
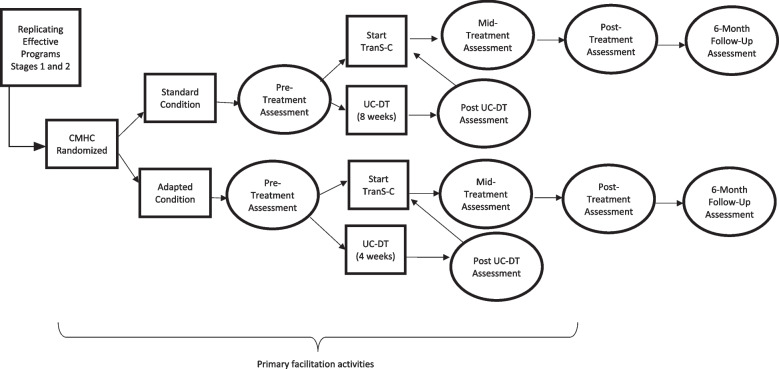
Community mental health center (CMHC) randomization and patient timeline

**Fig. 2:**
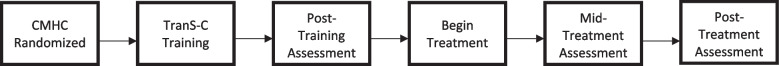
Provider timeline

This study was preregistered on clinicaltrials.gov (identifier: NCT04154631) and received approval from the Committee for the Protection of Human Subjects at the University of California, Berkeley. Any protocol changes will be submitted to clinicaltrials.gov and the Committee for the Protection of Human Subjects. The research team will communicate relevant changes to the CMHCs and in appropriate publications (e.g., see subsection on Changes to Preregistration below). If there are too many findings to reasonably interpret in one paper, we may separate some of the findings into two or more papers. This research is funded by the National Institute of Mental Health (R01MH120147). The present protocol used the SPIRIT reporting guidelines [52] (see SPIRIT checklist in Additional file 1 and Figs. 1 and 2).

### Facilitation

In the present research, facilitation was selected as the core implementation strategy used to implement TranS-C in the CMHCs, based on promising evidence [53–55]. Facilitation has been defined as “multi-faceted interactive process of problem solving, enabling and supporting individuals, groups, and organizations in their efforts to adopt and incorporate innovations into routine practices” [56]). This approach is grounded in the Promoting Action on Research Implementation in Health Services (PARiHS) framework [57]. In practice, each CMHC receives direct support from the lead facilitator, who is a licensed clinical social worker with expertise in community mental health and sleep treatment (ERA), and a team of trained facilitators employed by the research team. Throughout the study, the facilitation team is supervised by the Principal Investigator (PI; AGH) with periodic check-ins with an REP and facilitation expert (AMK). Team activities are also informed by the Veterans Affairs facilitation manual [58] and Harvey and Kitson’s [57] Facilitation Guide. Additionally, the lead facilitator (ERA) and postdoctoral scholar (LDS) completed the Behavioral Health Veterans Affairs Quality Enhancement Research Initiative Implementation (BH QUERI) Facilitation Training and regularly attended BH QUERI’S monthly drop-in consultation group.

The overarching approach of the facilitators is to conduct ongoing assessments for each CMHC site and then plan responses to identify unmet needs and mitigate barriers. This is accomplished via integrated bundles of evidence-based implementation strategies informed by implementation science [59, 60].

The specific activities of the facilitators include the following. First, they organize and lead regular TranS-C trainings for interested CMHC providers. Second, the facilitators distribute treatment manuals and workbooks to all participating providers and patients. Third, drop-in supervision is offered once a week separately for each condition by the lead facilitator (ERA) and a clinical science graduate student (CAC) with expertise in TranS-C and SMI. Fourth, consultation is provided on an ongoing, as-needed basis by the facilitators via phone calls, text messaging, and emails. Fifth, the facilitators organize and lead regular presentations to the CMHCs on advanced topics related to sleep and mental health (e.g., Lunch & Learn, Coffee Colloquium, Booster Sessions). Sixth, facilitators help with administrative barriers, such as working to ensure that TranS-C trainings count toward Continuing Education credits. Seventh, facilitators offer a sleep treatment certification that CMHC providers can achieve via three supervised TranS-C cases. Eighth, facilitators maintain an active website with supplemental treatment-related resources for CMHC providers. Ninth, facilitators develop and distribute materials (e.g., flyers, educational videos, social media posts) related to sleep knowledge and mental health at the request of CMHC leadership and providers. Tenth, facilitators hold regular and as-needed meetings with CMHC leadership and key providers to provide progress updates, collaborate on decision-making, and problem-solve barriers to effective intervention delivery such as organizational burnout, site cultures that are resistant to change, lack of resources or funding, oversaturation of treatment options, and logistical barriers. Eleventh, facilitators identify and cultivate intervention “champions” who can model effective treatment delivery and support their colleagues in using Trans-C.

### Participants

Participants in the present study are drawn from CMHCs and consist of CMHC providers and CMHC patients 1. The inclusion criteria for selecting the CMHC sites within counties from which to recruit providers and patients are as follows: (1) provision of publicly funded adult mental health outpatient services and (2) support from CMHC leadership.

CMHCs preferred to determine which providers are eligible to receive TranS-C training at each site (e.g., case managers, nurses, psychiatrists), because this aligns with their real-world practice. The other inclusion criteria for providers are as follows: (1) employed or able to deliver client-facing services to patients within the CMHC, (2) interest in learning and delivering TranS-C, and (3) volunteer to participate and formally consent to participate.

The inclusion criteria for patients are as follows: (1) aged 18 years and older; (2) meet criteria for an SMI per self-report and confirmed by referring provider or administration of the Mini International Neuropsychiatric Interview (DSM-5, Version 7.0.0) by a licensed clinical social worker on the research team; (3) exhibit a sleep or circadian disturbance as determined by endorsing 4 (quite a bit) or 5 (very much), or the equivalent for reverse-scored items, on one or more PROMIS-Sleep Disturbance questions [61, 62]; (4) guaranteed place to sleep for at least 2 months that is not a shelter; (5) receiving the standard of care for the SMI and consent to regular communications between the research team and provider; and (6) consent to access their medical record and participate in assessments.

Patients will be excluded if they meet any of the following criteria: (1) presence of an active and progressive physical illness or neurological degenerative disease that is directly related to the onset and course of the sleep and circadian problems, or making participation in the study unfeasible, as assessed by the Checklist of Medical Conditions and Symptoms on the Duke Structured Interview for Sleep Disorders [63] and clinical interview; (2) presence of substance abuse/dependence only if it makes participation in the study unfeasible; (3) current active intent or plan to commit suicide (those with suicidal ideation are eligible) only if it makes participation in the study unfeasible, or homicide risk; (4) night shift work for more than two nights per week in the past 3 months (i.e., regularly scheduled work from 12 a.m. to 6 a.m.); or (5) pregnant or breastfeeding.

### Recruitment

#### Community mental health centers

Building the CMHC network that forms the basis for this study began in August 2013 with outreach by the PI. The network has been maintained via newsletters, meetings, and workshops on EBPTs. Originally, eight counties agreed to participate. At various stages of the study, we have continued to focus on recruiting new counties and new CMHC sites to maximize provider and patient sample size goals. Most counties consist of three to 10 CMHC sites. Sites in the following ten counties in California, USA, are currently participating in the Implementation Phase: Alameda, Contra Costa, Kings, Monterey, Placer, Santa Cruz, Solano, Santa Clara^2^, Santa Barbara, and Lake. Note that sites in San Louis Obispo are also participating but are operating as part of Monterey County.

#### Providers

Facilitators meet with key CMHC leadership, who help to engage and recruit providers in their CMHC. In some CMHCs, this involves leadership requiring all staff to complete the TranS-C training, whereas in other CMHCs, leadership advertises the opportunity and allows anyone who is interested to register. During the TranS-C trainings, facilitators continue to engage and recruit providers by describing the benefits of participating in the study. These benefits include possible improvement in sleep and mental health for patients, certification in TranS-C for providers, expert consultation from the UC Berkeley research team, hard copies of the treatment materials, enrollment prizes, and financial compensation received by participating patients. After TranS-C trainings, facilitators follow up with weekly emails for 1 month that highlight each of these benefits and present other resources related to TranS-C, sleep, and mental health. Providers are also recruited through flyers posted in CMHCs, announcements at staff meetings, meetings organized by the facilitators, and appointments by leadership. Strategies to maintain relationships with providers and optimize data collection are ongoing by facilitators, including workshops and trainings, “enrollment challenges” and prizes (e.g., treatment-related books, magnets, t-shirts, mugs, and gift cards), continuing education credits for participation, and distributing newsletters or other topical resources.

#### Patients

Patients are recruited through a variety of methods, based on each CMHC’s preference. These methods include the following: (1) posting flyers from the research team in waiting rooms and providers’ offices; (2) integrating a sleep screener into intake paperwork; (3) asking providers to screen patients on their caseload; and (4) encouraging word of mouth between patients. Potentially eligible patients are typically identified by their provider. With the patient’s consent, the provider contacts the facilitators, who connect the patient with the assessment team so that the patient can be formally evaluated for eligibility and enrolled in the study. After eligibility has been confirmed and consent to participate in the study has been given, the patient is matched to a CMHC TranS-C provider. Ideally, the TranS-C provider is the patient’s own provider (e.g., their case manager, nurse, physician). If this is not possible, an alternative provider is identified. Patient retention is maximized via collaborative efforts between the providers, facilitators, and assessment team. Considerable efforts are made by the facilitators and assessors to answer questions and troubleshoot challenges (e.g., scheduling difficulties) to prevent attrition.

### Interventions

Two variations of TranS-C are tested in the present research: Standard TranS-C and Adapted TranS-C. Both are delivered alongside the usual care offered by each CMHC. The control condition is usual care followed by delayed treatment with Adapted or Standard TranS-C (UC-DT). In the CMHCs, usual care consists of working with a service provider (e.g., psychologist, case manager, occupational therapist, psychiatrist, nurse practitioner) who provides direct mental health support from within their scope of practice. The patient might also be referred by that provider for other services as needed (e.g., healthcare, housing support, nutrition, vocational specialists, or peer advocacy). Occasionally patients receive treatment from interdisciplinary or residential teams, meaning their services are coordinated across multiple service providers. Although most providers delivered TranS-C via individual sessions, some chose to deliver it in a group setting. Note that TranS-C was originally developed in English, then translated into Spanish about four months into data collection to expand access and subsequently offered by Spanish-speaking providers. The treatment conditions, along with the adaptation process for Adapted TranS-C, are described below.

#### Standard TranS-C

Standard TranS-C is delivered by CMHC providers across eight 50-min, weekly sessions [40]. It is comprised of 4 cross-cutting modules featured in every session, 4 core modules, and 7 optional modules that are used based on clinical presentation, treatment goals, and provider case conceptualization. The *cross-cutting modules* are case formulation, sleep and circadian education, motivational enhancement, and goal setting. *Core module 1* targets irregular sleep–wake times, difficulty winding down, and difficulty waking up. *Core module 2* aims to reduce daytime impairment*. Core module 3* focuses on unhelpful beliefs about sleep*. Core module 4* aims to promote maintenance of changes made during treatment. *Optional module 1* addresses poor sleep efficiency via stimulus control [64] and sleep restriction [65]. *Optional module 2* helps patients reduce time in bed. *Optional module 3* addresses delayed or advanced phase problems (e.g., going to sleep later than desired or waking up earlier than desired). *Optional module 4* helps patients manage worries about sleep. *Optional Module 5* promotes compliance with Continuous Positive Airways Pressure (CPAP) for patients with sleep apnea. *Optional Module 6* helps patients negotiate sleep in complicated environments (e.g., noise from bed/roommates, traffic noise, streetlight entering the bedroom). *Optional Module 7* is for patients who experience nightmares. Training for the Standard TranS-C condition consists of a 1-day workshop (i.e., 6–8 h) or two, 3-h training blocks.

#### Adapted TranS-C

Adapted TranS-C is delivered by CMHC staff across four, 20-min, weekly sessions. Treatment consists of the same four *cross-cutting and core modules* as in Standard TranS-C, but the core modules are split up into five, rather than four, modules. *Core module 1* targets irregular sleep–wake times. *Core module 2* targets difficulty winding down. *Core module 3* targets difficulty waking up. *Core module 4* aims to reduce daytime impairment. *Core module 5* promotes the maintenance of change. The one *optional module* focuses on reducing sleep-related worry and can be integrated with the core modules, based on clinical presentation, treatment goals, and provider case conceptualization. Training for the Adapted TranS-C condition consists of four, 1-h workshops or two, 2-h workshops, based on CMHC preferences.

There have been calls for rigorous approaches to treatment adaptation [66, 67]. In response, we grounded the process for adapting TranS-C in theory, data, and stakeholder input. As the overarching guide for the adaptation process, the Replicating Effective Programs (REP) framework [47] was used. Phase 1 of REP (Pre-Condition) was completed prior to the present protocol. First, as discussed above, we established that there is a need for effective, feasible EBPTs for SMI in CMHCs and that sleep and circadian functioning may represent a powerful target to help address this need. Second, we determined that there was empirical support for TranS-C in CMHCs [35]. Third, we gathered stakeholder input on fit and packaging of the intervention [46, 68]. Fourth, we reviewed past data and identified the TranS-C treatment skills that were most utilized by patients with a utilization scale adapted from [69]. Fifth, we considered TranS-C’s theoretical underpinnings and mechanisms of action [40, 41] from which we retained the core elements [66, 70]. Sixth, we piloted Adapted TranS-C with 21 adults through the PI’s UC Berkeley research clinic (unpublished data). Informal feedback was solicited from providers and patients who participated in this pilot to further refine Adapted TranS-C. In phase 2 of REP (Pre-Implementation), we customized the delivery of TranS-C training and treatment materials to the CMHC context based on the input from CMHC leadership, staff, and patients [46, 68]. Throughout REP phases 1 and 2, following leading adaptation frameworks, we sought to ensure that Adapted TranS-C would be relevant to the broadest range of patients and to account for factors that impact implementation (e.g., resources required) [66, 71, 72]. The present trial will address the last two phases of REP—namely, phases 3 (Implementation) and 4 (Maintenance and Evolution).

#### UC-DT

In UC-DT, patients begin with usual care for 4 weeks if their CMHC is randomized to Adapted TranS-C and 8 weeks of usual care if their CMHC has been randomized to Standard TranS-C. After the delay, they receive Adapted or Standard TranS-C, also based on the condition to which their CMHC has been randomized (see Fig. 1). The decision to compare TranS-C to UC-DT was made in close collaboration with the early CMHC partners. This design aims to strike a balance between (a) including a comparison group to demonstrate the effectiveness of TranS-C in community settings; (b) ensuring that *all* participants receive what we hypothesize to be an active treatment (TranS-C); and (c) maximizing efficiency in terms of study duration, budget, and participants’ time investment. Notably, usual care has been the comparison group in several influential studies [73–75].

### Measures

In addition to the measures below, a sociodemographics form is completed by providers and patients. Only measures that will be analyzed for the primary aims of the Implementation Phase (see above) are reported below. See Table 1 for timing of each measure.

**Table 1:** SPIRIT depiction of timing and measures collected for implementation phase

	**Screening**	**Post-training**	**Pre-treatment**	**Mid-treatment**	**Post-treatment**	**6 months post-treatment**
**Patient**						
Sociodemographics			x		x	x
Eligibility Items	x					
PROMIS-SD^P^	x		x	x	x	x
PROMIS-SRI			x	x	x	x
DSM-5 Cross-Cutting			x		x	x
SDS			x		x	x
Sleep Health Composite			x	x	x	x
PHENX Toolkit			x		x	x
CEQ					x	
**Provider**						
Sociodemographics		x				
Occupation		x				
Acceptability^P^		x		x	x	
Appropriateness		x		x	x	
Feasibility		x		x	x	
Number of Sessions					x	

Allocation to Adapted or Standard TranS-C occurs at the county level and prior to enrollment of any participants in that county (i.e., patients or providers). Enrollment of patients and allocation to immediate TranS-C or delayed TranS-C (UC-DT) occur after the screening and before the pre-treatment assessment. Enrollment of providers occurs after the training. ^*P*^, primary outcome; *PROMIS-SD*, PROMIS-Sleep Disturbance—note: PROMIS-SD is only assessed during the pre-treatment assessment if done more than one month after the screening to minimize the burden for patients; *PROMIS-SRI*, PROMIS-Sleep Related Impairment; *SDS*, Sheehan Disability Scale; *CEQ*, Credibility/Expectancy Questionnaire

#### Providers

##### Primary outcome

*Acceptability*: Providers rate the acceptability of TranS-C via the *Acceptability of Intervention Measure* [76]. This 4-item measure is rated on a scale from 1 (completely disagree) to 5 (completely agree). This measure has demonstrated satisfactory known-groups validity, internal reliability, test–retest reliability, and sensitivity to change [76].

##### Secondary outcomes

*Appropriateness and feasibility*: Providers rate the appropriateness and feasibility of TranS-C via the following 4-item measures: *Intervention Appropriateness Measure* and *Feasibility of Intervention Measure* [76]. Both measures are rated on a scale from 1 (completely disagree) to 5 (completely agree). These measures have demonstrated satisfactory known-groups validity, internal reliability, test–retest reliability, and sensitivity to change [76].

##### Other measures

*Number of TranS-C Sessions*: The number of sessions delivered to each enrolled patient by each provider will be counted.

*Occupation*: Providers are asked to report their current position, professional degree, and work history, including their caseload, theoretical orientation, licensure status, and previous training in sleep treatment.

#### Patients

##### Primary outcome

*Sleep disturbance*: The 8-item PROMIS-Sleep Disturbance (PROMIS-SD) assesses disruption to sleep (e.g., restlessness, trouble staying asleep) over the past 7 days [62]. Items are rated on a scale from 1 (not at all/never/very poor) to 5 (very much/always/very good), and scores range from 8 to 40, with higher scores indicating greater disturbance. This measure has demonstrated acceptable reliability and validity [61, 62].

##### Secondary outcomes

*Sleep-related impairment*: The 16-item PROMIS-Sleep Related Impairment (PROMIS-SRI) assesses daytime impairment related to sleep problems over the past 7 days on a scale from 1 (not at all/never) to 5 (very much/always) [62]. Scores range from 16 to 80, with higher scores indicating greater impairment (e.g., daytime sleepiness, difficulty concentrating). This measure has demonstrated excellent psychometric properties [61, 62].

*Functional impairment*: Functional impairment is assessed via the Sheehan Disability Scale (SDS) [77]. Impairment in work and school, social life, and home and family is rated on a scale from 0 (not at all) to 10 (extremely). Scores range from 0 to 30, with higher scores indicating greater impairment. This measure has demonstrated good reliability and validity [77, 78].

*Overall sleep health*: The Sleep Health Composite is proposed to capture overall sleep health for the complexity of sleep problems in SMI that are covered by TranS-C [79]. It is defined as the sum of scores on six sleep health dimensions (each dimension dichotomized as 1 = good; 0 = poor): regularity (midpoint fluctuation), timing (mean midpoint), efficiency (sleep efficiency), duration (total sleep time), satisfaction (sleep quality question on PROMIS-SD), and alertness (daytime sleepiness question on PROMIS-SRI). All dimensions—except satisfaction and alertness—are assessed via questions about sleep–wake patterns over the past 7 days (e.g., *In the past week, what time have you usually woken up in the morning?*). Scores range from 0 to 6, with higher scores indicating better sleep health. Initial validity of this measure has been established [79].

*Psychiatric symptoms*: The DSM-5 Cross-Cutting Measure assesses psychiatric symptoms across 13 mental health domains. Participants rate how often they were bothered by each symptom on a scale from 0 (not at all) to 4 (nearly every day). Scores range from 0 to 52, with higher scores indicating more severe symptoms. This measure has demonstrated good test–retest reliability and clinical utility [80, 81].

##### Exploratory outcomes

*PhenX Toolkit: substance use and suicidality*—Scales from the PhenX Toolkit [82] are used to assess various patient outcomes, including suicidal ideation and behaviors, alcohol, tobacco, and other psychoactive substances (e.g., cannabis, hallucinogens, sedatives). PhenX measures have been compiled by working groups and domain experts via a consensus process to facilitate consistency across studies [82]. To assess suicidal ideation and behaviors, the PhenX “Classification of Suicidal Ideation and Suicidal Behavior—Adult – Current” protocol is used. This protocol includes two subscales from the screening version of the Columbia-Suicide Severity Rating Scale: Severity of Suicidal Ideation and Suicidal Behavior, assessing suicidality during two time periods—namely ideation in the past month and suicidal behavior in the past three months. To ease the patient burden, this measure was adapted slightly, such that if patients deny suicidal ideation, they are not required to answer questions about suicidal behavior. To assess alcohol, the PhenX “Alcohol—30-Day Quantity and Frequency” protocol is used. This protocol measures both quantity and frequency of alcohol consumption. To assess tobacco, the PhenX “Tobacco—30-Day Quantity and Frequency—Adult” protocol is used. This measure has three sets of question protocols: (1) a protocol for “Every-Day Smokers,” (2) a protocol for “Some-Day Smokers,” and (3) a protocol for “Former Smokers.” If patients report that they have never smoked tobacco, this measure is skipped. To assess the use of substances and other drugs, the PhenX “Substances—30-Day Frequency” protocol is used. This measure assesses use of substances such as sedatives, painkillers, stimulants, and hallucinogens. In addition, caffeine is assessed using questions adapted from the “Supplemental Beverage Questionnaire.” Questions used in the present study assess frequency and quantity of caffeinated or decaffeinated drinks consumed over the past 30 days.

*Credibility and perceived improvement*: Perceptions of TranS-C credibility and symptom improvement are assessed by four questions adapted from the Credibility/Expectancy Questionnaire (CEQ) [83]. These questions assess (1) how logical TranS-C seemed, (2) how successful it was in reducing sleep symptoms, (3) how confident patients would be in recommending TranS-C to a friend, and (4) how much improvement patients believe had occurred. All questions are rated on a scale from 0 (not at all) to 9 (very), except for expected improvement, which is rated as a percentage from 0 to 100%.

### Procedure

Providers and patients are consented by the assessment team prior to participation. All participants are informed that they can withdraw from the study at any time. All patients are compensated for their participation, and providers are compensated if permitted by their CMHC. The assessments are completed by the assessment team, comprised of experienced assessors. Note that assessors complete the consent process to minimize burden on participants (e.g., this practice reduces number of calls from team). Because the assessors need to provide study-related information—such as number of assessments and treatment sessions—to the patients during the consent process, the assessors are not blind to condition at pre-treatment. However, at post-treatment and 6FU, we endeavor to keep assessors blind to condition. As is common in clinical trials, there are ways that assessors may be able to infer treatment condition (e.g., slightly different assessment batteries, patients may ask assessors “when does treatment start?” during the post-delay assessment). Assessors receive ongoing supervision and are thoroughly trained to deliver the surveys with integrity and minimal bias.

#### Providers

Provider assessments are completed after TranS-C training, at mid-treatment, and at post-treatment. See Fig. 2 for the provider timeline.

#### Patients

Patient assessments in the immediate TranS-C treatment conditions are completed at pre-treatment, mid-treatment, post-treatment, and 6 months after treatment (6FU). Patient assessments in the UC-DT condition are completed at pre-treatment and 4 or 8 weeks after pre-treatment (i.e., post UC-DT), depending on whether their county has been randomized to Adapted or Standard TranS-C, respectively. After the post-UC-DT assessment, patients start delayed treatment with TranS-C. They subsequently complete assessments at mid-treatment, post-treatment, and 6FU. Note that patients do not complete a 6FU assessment after the delayed portion of UC-DT. This was a compromise made with CMCH partners, so that patients would not need to wait 7–8 months to receive treatment. See Fig. 1 for the patient timeline.

### Allocation

CMHCs and patients are randomized through a computerized randomization sequence. We do not stratify during randomizing at the county level. When randomizing patients, we stratify for the presence of psychosis or not (current), presence of substance use or not (current), and age (≥ 50 or not), as there is evidence that these variables can impact sleep or treatment outcome [50, 84, 85]. Only the facilitators, assessors, and research team (i.e., not CMHCs, providers, or patients) are privy to which CMHCs and patients are allocated to which TranS-C treatment condition (Adapted versus Standard TranS-C). CMHC providers and patients know whether their patients have been randomized to receive immediate or delayed treatment. The facilitator informs each provider when they can start having sessions. In the immediate condition, the provider is asked to begin sessions as soon as possible. In the delayed condition, the provider is asked to wait until after the patient has completed the post-delay assessment (i.e., approximately 4 weeks in the Adapted condition or 8 weeks in the Standard condition).

### Sample size

Sample size was determined via power analyses with Optimal Design [86, 87] and Stata 15 [88] and a two-sided alpha of 0.05. We used the original *N* = 8 counties that agreed to participate, though as noted above, new counties have since been recruited to meet sample size goals determined by the following calculations. For Aims 1 and 3, 60 patients from eight randomized clusters/CMHCs were found to provide over 90% power to detect large effects (average *d* = 0.89) between TranS-C and UC-DT [35], and over 80% power to detect a medium effect (*d* = 0.50) between Standard and Adapted TranS-C. For Aim 2, based on a prior study with a similar aim/measure [89], we expected a large effect size (*d* = 0.80) between Standard and Adapted TranS-C on the primary outcome of acceptability. Prior studies have reported high correlation coefficients between acceptability, feasibility, and appropriateness (*r*s = 0.67–0.90) [76, 90]; thus, the analysis for acceptability was expected to sufficiently power the other outcomes. Based on the site intra-class correlation (ICC) estimated from similar prior studies [91, 92], we assumed the ICC to be 0.01 and the inflation factor was calculated as 1 + (*n* − 1) × ICC. Eight clusters with an average cluster size of 10 were found to be needed to achieve over 80% power to detect a large effect for the outcome (acceptability) at the provider level. Thus, to test this aim, we would need at least 80 CMHC providers: 10 providers from 8 CMHCs (at least 4 CMHCs per randomized arm).

In sum, we aimed to randomize at least eight CMHC clinics to either Standard or Adapted TranS-C, with at least 80 providers (10 per CMHC). The goal for patients is 240 patients (30 per CMHC) randomized to immediate Standard or Adapted TranS-C and 240 randomized to UC-DT (30 per CMHC). Adding 20% to account for attrition, the total target sample size for providers is 96 and for patients is 576.

### Data management and dissemination

All patient-identifiable data are saved by the assessment team on password-protected fillable PDFs on a secure password-protected and HIPAA-compliant website. On these PDFs, patients and providers are assigned identification numbers. These identification numbers are then used to link anonymized data that is collected via password-protected Qualtrics. When collecting assessments, assessors call participants and enter the data into Qualtrics. Participants also have the option of entering their data directly into a participant-facing version of the surveys via a HIPAA-compliant version of Qualtrics. Patient-identifiable data is not shared with outside entities during or after the trial. A data management team supervised by the PI, biostatistician (LD), and postdoctoral scholar (LDS) is responsible for downloading, collating, and analyzing the data.

A Data Safety Monitoring Board has been formed to help prevent and manage adverse events. The board includes members with expertise in SMI, psychosocial treatments, and randomized controlled trials. Members are independent from the PI and competing interests. A report was made to the board bi-annually for the first year of the research. Since then, it has shifted to annual reports. However, if safety issues arise, it will be changed to monthly meetings. Yearly reports are submitted to the Committee for the Protection of Human Subjects at UC Berkeley and National Institute of Mental Health.

Outcomes specifically of interest to our partners are presented to CMHC leadership as part of the widely used implementation strategy, audit, and feedback [93]. However, these interim analyses are used only for facilitation purposes. In other words, they do not influence research procedures in any way (e.g., to inform when to terminate the trial).

Results from the trial, as well as analysis code, will be shared via peer-reviewed publications, professional conference presentations, and meetings and newsletters to CMHCs, as relevant. Other than the authors and compliance with data-sharing agreements stipulated by the National Institutes of Health, no other entities have contractual agreements to access the final dataset. Deidentified data are submitted to the National Institute of Mental Health Data Archive twice per year, per their requirements.

### Roles and responsibilities

This trial is supervised by the PI (AGH), who manages the facilitation team, assessment team, and the data management team. The PI meets with members of each team as needed in addition to daily email communication. Within each team, there is at least one trained lead (ERA, KF, JMS, LD, LDS) who supervises the day-to-day activities of other team members. There is no coordinating center, trial steering committee, or Stakeholder and Public Involvement Group. The responsibilities of each team are detailed elsewhere in this protocol. In summary, the facilitators execute the implementation of TranS-C via numerous activities, including training and supervising CMHC providers in the delivery of TranS-C. The assessment team is responsible for the informed consent process and conducting participant (i.e., provider and patient) evaluations. CMHC leadership and enrolled providers work with the facilitation team to recruit additional providers. CMHC providers help to identify potentially eligible patients, who are then connected with the assessment team for formal eligibility evaluation.

### Changes to preregistration

In December 2022, updates relevant to the present protocol were made to the clinicaltrials.gov protocol (identifier: NCT04154631). These updates can be summarized as follows. First, minor changes were made to four inclusion and exclusion criteria, based on feasibility and preferred practices of CMHC partners. For instance, as described above, CMHCs preferred to determine which providers were eligible to deliver TranS-C, and thus, providers held a wider range of positions than we had originally anticipated. Additionally, in rare cases, providers outside of CMHCs participated in the study (e.g., a supervisee from a CMHC provider’s private practice participated). Thus, we changed the provider criteria to be more inclusive (see provider inclusion criterion 1 above). Second, as the study unfolded, rich data emerged. To maximize the resources invested in this study, we decided to systematically capture these data with additional measures, now included in the “Other Measures” section of clinicaltrials.gov. For example, the number of sessions delivered to each enrolled patient per provider was already being logged by the facilitators. These data will be included in the dropout calculations and sensitivity analyses of the Implementation Phase main aims (see the “Measures” and “Planned analyses” sections). Third, as described above, to maximize recruitment goals, additional CMHC sites were recruited from new counties on an ongoing basis. We have added these additional sites/counties to clinicaltrials.gov. Fourth, the sample size has been changed to match the power analysis described above. The sample size in the original clinicaltrials.gov preregistration reflected a request from one reviewer of the National Institute of Mental Health grant to increase the sample size to account for a higher rate of dropout. However, this was deemed to be unfeasible, and the recruitment goal was adjusted to reflect the power analysis. Fifth, the type of design was corrected to be hybrid type 2, given the equal emphasis on determining effectiveness of an intervention (i.e., TranS-C) and feasibility/impact of an implementation strategy (i.e., adapting interventions to fit context) [48, 49]. Sixth, after launching the study, we received requests to minimize burden for patients. In response, assessors began consenting patients during pre-treatment assessments. Thus, we now specify that assessors are blind to condition at post-treatment and 6FU.

## Planned analyses

### Preliminary analyses and missing data

Analyses will use all available data (intent-to-treat) [94]. If dropout is related to other variables, they will be included as predictors. Baseline between-group differences on demographic variables will be examined. These tests will not be used to select covariates in the primary intention-to-treat analysis [95–98]. Instead, covariates will be carefully selected at the conclusion of the trial—given the variations due to COVID-19 and the CMHC context (see the “Discussion” section)—and the potential influences of baseline differences will be evaluated as moderators (approach to moderation described below). Analyses comparing TranS-C to UC-DT will evaluate change in outcomes from pre-treatment to post-treatment. Analyses comparing Adapted to Standard TranS-C will evaluate change in outcomes from pre-treatment to post-treatment and pre-treatment to 6FU (see the “Method” and “Discussion” section for more details).

Distributions will be evaluated to detect outliers, and we will ensure that assumptions of planned analyses are met. Covariates will include the patient variables for which we stratified (i.e., age and presence of psychosis or substance use). For all statistical models, counties will be entered as dummy variables rather than a level of analysis due to the relatively small number of clusters. The average intraclass correlation on provider and patient-level outcomes will be reported. The Benjamini–Hochberg procedure [99] will be used to correct for multiple testing for confirmatory analyses on the primary outcomes (i.e., sleep disturbance and acceptability) [100].

### Dropout

The *N* by stage of dropout will be reported for the following: dropout after randomization but before the first treatment session, dropout after treatment has begun but attended half or fewer of the intended number of sessions (i.e., ≤ 2 in Adapted, ≤ 4 in Standard), dropout after attended more than half the intended number of sessions (i.e., > 2 in Adapted, > 4 in Standard) but before treatment has been completed, and dropout after treatment has been completed but prior to post-treatment or 6FU assessments. The number of patients who completed a post-treatment assessment but were lost to 6FU will also be reported. When available, the reasons for dropout and improvement among patients who drop out will be reported.

### Aim 1: Effectiveness outcomes of Standard or Adapted TranS-C versus UC-DT

Multilevel modeling (MLM) [101–103] will be used to account for multiple observations nested within patient. The level 1 equation will include dummy-coded time indicators as the predictor (0 = pretreatment, 1 = post-treatment). The level 2 equation will include dummy-coded treatment condition (0 = UC-DT, 1 = Adapted or Standard TranS-C), treatment-by-time interaction terms, and dummy-coded counties (0 = Alameda, 1 = Contra Costa, 2 = Kings, 3 = Monterey, 4 = Placer, 5 = Santa Cruz, 6 = Solano, 7 = Santa Clara, 8 = Santa Barbara, and 9 = Lake) as predictors. The treatment effects of interest will be significant treatment-by-time interactions at the 5% level on the primary outcome of sleep disturbance and the secondary outcomes of sleep-related impairment, functional impairment, and psychiatric symptoms, all modeled as continuous variables. Significant treatment-by-time interactions indicate that change in patient outcomes is significantly different comparing Adapted or Standard TranS-C to UC-DT. Significant interactions will be interpreted using planned contrasts (i.e., treatment effects on change from pre-treatment to post-treatment) and graphs. Additionally, the indirect effects of TranS-C relative to UC-DT on functional impairment and psychiatric symptoms through improvements in sleep disturbance and sleep-related impairment will be tested using multilevel structural equation modeling [104].

### Aim 2: Adapted TranS-C versus Standard TranS-C on fit to CMHC context

MLM will be used to account for multiple observations nested within providers. TranS-C treatment condition (Adapted versus Standard TranS-C) will be evaluated as a predictor of fit, operationalized as provider ratings of acceptability, feasibility, and appropriateness. The level 1 equation will include dummy-coded time indicators as the predictor (0 = post-training, 1 = post-treatment). The level 2 equation will include dummy-coded treatment condition (0 = Standard TranS-C, 1 = Adapted TranS-C), treatment-by-time interaction terms, and dummy-coded counties (0 = Alameda, 1 = Contra Costa, 2 = Kings, 3 = Monterey, 4 = Placer, 5 = Santa Cruz, 6 = Solano, 7 = Santa Clara, 8 = Santa Barbara, and 9 = Lake) as predictors. The treatment effects of interest will be significant treatment-by-time interactions at the 5% level on the primary outcome of acceptability and the secondary outcomes of feasibility and appropriateness, all modeled as continuous variables. Significant treatment-by-time interactions indicate that change in perceptions of fit is significantly different comparing Adapted to Standard TranS-C. Significant interactions will be interpreted using planned contrasts (i.e., treatment effects on change from post-training to post-treatment) and graphs.

### Aim 3: Fit as a mediator of treatment condition and patient outcome

Multilevel structural equation modeling will be used to test whether improved perceptions of fit (i.e., acceptability, appropriateness, and feasibility) mediate the relation between TranS-C treatment condition (i.e., Adapted versus Standard TranS-C) and change in the primary patient outcome of sleep disturbance and the secondary patient outcomes of sleep-related impairment, functional impairment, and psychiatric symptoms. Models will evaluate change in outcomes from pre-treatment to post-treatment and pre-treatment to 6FU.

### Sensitivity analyses

Three sets of sensitivity analyses will be run to help account for the complexities of the COVID-19 pandemic and the CMHC context. In the first set, the analyses for Aims 1–3 will be conducted with (a) treatment completers, (b) patients who completed more than half the number of the suggested sessions (i.e., > 2 sessions for Adapted and > 4 sessions for Standard), and (c) patients who completed half or fewer of suggested sessions. In other words, these analyses will test the effectiveness of TranS-C at varying doses, which may be important considering evidence on “early responders” [105] and “real world” contexts where turnover and dropout can be high [106, 107]. In the second set of sensitivity analyses, we will assess whether any patients who did not complete post-treatment or 6FU had achieved meaningful clinical improvement by mid-treatment, using a reliable change index for the primary outcome of PROMIS-SD [108]. For the sensitivity analyses, we will define these patients as completers, and we will use their mid-treatment assessment in place of a post-treatment assessment. Then, all pre- to post-treatment analyses for Aims 1–3 will be rerun. In the third set of sensitivity analyses, we will run the analyses for Aims 1–3 but only include post-treatment and 6FU assessments that were collected within 3 months of the target assessment date (e.g., a 6FU assessment that was completed nine months after treatment ended).

### Exploratory Aim 1: TranS-C treatment condition on credibility and PhenX Toolkit

MLM will be used to test TranS-C treatment condition (Adapted vs. Standard TranS-C) predicting PhenX Toolkit outcomes of substance use and suicidality at post-treatment and 6FU. The approach to MLM will mirror Aim 2, except the outcomes will be substance use and suicidality from the PhenX Toolkit. Linear regression will be used to test treatment condition (Adapted vs. Standard TranS-C) predicting patient perceptions of TranS-C’s credibility and perceived improvement at post-treatment.

### Exploratory Aim 2: Treatment effects moderated by risk factors

Using MLM, three-way interactions between treatment condition (Adapted or Standard TranS-C versus UC-DT), time, and risk factors will be used to evaluate moderators of patient outcome (i.e., sleep and circadian problems, functional impairment, and psychiatric symptoms). Each moderator and outcome will be tested in a separate model. Moderators will include age, sex, and sleep/circadian and psychiatric symptoms at baseline. The level 1 equation will include the moderator and dummy-coded time indicators as the predictors (0 = pre-treatment, 1 = post-treatment). The level 2 equation will include dummy-coded treatment condition (0 = UC-DT, 1 = Adapted or Standard TranS-C), treatment-by-time by moderator interaction terms, and dummy-coded counties (0 = Alameda, 1 = Contra Costa, 2 = Kings, 3 = Monterey, 4 = Placer, 5 = Santa Cruz, 6 = Solano, 7 = Santa Clara, 8 = Santa Barbara, and 9 = Lake) as predictors. A significant interaction indicates a moderating effect and will be probed with planned contrasts (e.g., moderating effects on the differences between treatments in change from pre-treatment to post-treatment) and graphs.

## Discussion

This study aims to evaluate the implementation and effectiveness outcomes of the Transdiagnostic Intervention for Sleep and Circadian Dysfunction (TranS-C) delivered to patients in community mental health centers (CMHCs) by CMHC providers. Findings will address several research priorities. First, TranS-C was designed to target a wide range of serious mental illness (SMI) diagnoses through addressing a wide range of sleep and circadian problems [35, 40]. As such, TranS-C has the potential to improve symptoms of patients with diverse and complex clinical presentations, which may help alleviate provider burden and optimize patient care [18, 19]. Second, providers play a pivotal role in the day-to-day delivery of mental health care. Thus, it is critical to assess their perceptions of implemented treatments. The present findings will advance our understanding of providers’ perceptions of TranS-C’s “fit”—namely, acceptability, feasibility, and appropriateness—which is an important predictor of implementation outcomes (e.g., fidelity, reach, sustainability) [15–17]. Third, leaders in the implementation science have advocated for causal tests of implementation strategies [109]. Heeding this call, the present study tests whether adapting TranS-C to CMHCs improves “fit” between the treatment and context, and if so, whether this improved fit is associated with better patient outcomes. Evaluating this implementation strategy (i.e., adapting treatments to fit contexts) may help other implementation scientists evaluate whether it is a worthwhile implementation strategy to pursue.

These potential contributions should be considered alongside the protocol’s methodological limitations. First, we did not collect data from leadership at our partner CMHCs. The primary reason was that these leaders have many demands on their time and already had generously collaborated with us on the design and implementation of the study. However, we recognize that leadership-level factors can meaningfully impact implementation outcomes [110]. Thus, exploring leadership perspectives on TranS-C will be an important direction for future research. Second, we considered additional assessment methods for sleep and circadian problems, such as a diagnostic interview and/or polysomnography. However, even without these more intensive diagnostic tools, some patients reported difficulty with the length of assessments. Additionally, we were interested in recruiting patients with a broad range of symptom severity, which may have more ecological validity than a more restricted sample. That said, we acknowledge that there are certain outcomes that we will not be able to address due to this design choice (e.g., change in diagnostic status). Third, assessors are not blind to treatment condition at pre-treatment. To minimize burden on participants (e.g., reduce number of calls from team), assessors are in charge of the consent process, and therefore, they need to provide study-related information—such as number of assessments and treatment sessions—to the patients. At post-treatment and 6FU, we endeavor to keep the assessors blind but, as is common in clinical trials, there are ways that assessors may be able to infer treatment condition (e.g., slightly different assessment batteries). In rare instances, patients in the UC-DT condition ask assessors questions such as “when does treatment start?” or “when will I receive the workbook?” during the post-delay assessment. In such instances, the assessor may be able to ascertain that the patient has been assigned to the UC-DT condition. Assessors receive ongoing supervision and are thoroughly trained to deliver the surveys with integrity and minimal bias. Additionally, relative to interviews or clinical rating scales, the standardized surveys delivered by assessors are likely less vulnerable to bias (see the “Measures” section). However, it is possible that assessor knowledge or hunches about patients’ condition may influence patient responses. Fourth, in the UC-DT condition, patients did not complete a 6FU assessment after the delay. This was a compromise made with CMCH partners, so that patients would not need to wait 7 to 8 months to receive treatment. Thus, analyses comparing TranS-C with UC-DT will evaluate outcomes from pre- to post-treatment, whereas analyses comparing Adapted versus Standard TranS-C can evaluate outcomes from pre- to post-treatment *and* pre- to 6FU. Depending on the outcomes from the trial, an important direction for future research will be testing the long-term effects of TranS-C relative to a control (e.g., UC-DT). Fifth, unforeseen challenges arose related to the COVID-19 pandemic and subsequent mandates in California (e.g., shelter-in-place). For some patients, sleep and circadian treatment became secondary to coping with other mental health ramifications of the pandemic (e.g., anxiety, isolation, grief) or even meeting basic needs of secure access to food and shelter [111]. Additionally, providers suddenly had more demands on their time, such as navigating the shift from in-person to virtual care—a shift that was also challenging for some patients. These changes had implications for the present study. For instance, we did not design the study to systematically evaluate potential differences in TranS-C delivery (i.e., in-person versus virtual versus phone). As another example, there was substantial turnover in providers and patients, leading to more discontinuity across treatment than anticipated. Sixth, some design choices were made to respect the expertise and preferences of our CMHC partners. For example, providers had the option to deliver TranS-C in a group or individual format. Even though this introduced variance into the study, it has been critical to the CMHCs, providers, and patients that we balance rigor with flexibility. We deliberated about whether to control for these variations necessitated by COVID-19 and the CMHC contexts. Ultimately, we decided against a prespecifying a comprehensive list of possible covariates, given the statistical drawbacks of controlling for many variables [95–98]. At the conclusion of the trial, the sources of variation that resulted from the COVID-19 pandemic and needs/preferences of our community partners will be carefully considered as to whether they should be included as covariates.

These challenges and limitations notwithstanding, in testing the implementation and effectiveness outcomes of TranS-C delivered by CMHC providers, this study has the potential to (a) expand support for a promising transdiagnostic treatment delivered to patients with SMI in routine practice settings, (b) take steps toward addressing some of the major challenges faced by providers in delivering evidence-based treatments (i.e., high caseloads, complex patients, poor fit), and (c) advance evidence on causal strategies in implementation science.

## Trial status

Protocol version 1, December 12, 2022. Data collection started in February 2020. Recruitment was completed in August 2022, but patient visits/assessments will continue through August 2023. Publishing of this protocol was delayed because of unforeseen challenges and uncertainties related to the COVID-19 pandemic and subsequent mandates (e.g., shelter-in-place), which began in California shortly after data collection started for this study.

### Supplementary Information

**Additional file 1.** SPIRIT checklist.

## Data Availability

Other than the authors and compliance with data-sharing agreements stipulated by the National Institutes of Health, no other entities have contractual agreements to access the final dataset. Deidentified data are submitted to the National Institute of Mental Health Data Archive twice per year, per their requirements.

## Abbreviations

TranS-C: Transdiagnostic Intervention for Sleep and Circadian Dysfunction
UC-DT: Usual care followed by delayed treatment with TranS-C
SMI: Serious mental illness
EBPT: Evidence-based psychological treatment
CMHC: Community mental health center
REP: Replicating Effective Programs
PARiHS: Promoting Action on Research Implementation in Health Services
PROMIS-SD: PROMIS-Sleep Disturbance
PROMIS-SRI: PROMIS-Sleep Related Impairment
PI: Principal investigator
ICC: Intraclass correlation
MLM: Multi-level modeling

## Footnotes

1 Note that the vast majority of providers and patients were employed by or seeking/receiving treatment at CMHCs. However, in very few isolated instances, providers and patients outside of CMHCs learned about the study (e.g., by word of mouth) and requested to participate. When the providers or patients otherwise met the criteria, they were permitted to participate and were matched with a CMHC patient or provider, respectively, by the facilitation team.

2 The site participating in Santa Clara is technically a federally qualified health center (FQHC), not a CMHC. Although there are some differences (e.g., funding structure, types of staff), these two types of sites are similar. Thus, for the purposes of this study, they are grouped together under “CMHC.”.
