# Correction: The Transdiagnostic Intervention for Sleep and Circadian Dysfunction (TranS‑C) for serious mental illness in community mental health part 1: study protocol for a hybrid type 2 effectiveness‑implementation cluster‑randomized trial

**DOI:** 10.1186/s13063-023-07479-7

**Published:** 2023-08-14

**Authors:** Laurel D. Sarfan, Emma R. Agnew, Marlen Diaz, Lu Dong, Krista Fisher, Julia M. Spencer, Shayna A. Howlett, Rafael Esteva Hache, Catherine A. Callaway, Amy M. Kilbourne, Daniel J. Buysse, Allison G. Harvey

**Affiliations:** 1 Department of Psychology, University of California, Berkeley, CA, Berkeley USA; 2 RAND Corporation, Santa Monica, CA USA; 3 University of Michigan and VA Ann Arbor Healthcare System, Ann Arbor, MI USA; 4 Department of Psychiatry, University of Pittsburgh, Pittsburgh, PA USA

**Correction: Trials 24, 198 (2023)**

**https://doi.org/10.1186/s13063-023-07148-9**

After publication of this article the authors spotted minor errors in the “**Planned Analyses**” section and (Table 1) of the original publication. As described in the original preregistration on clinicaltrials.gov (NCT04154631): the measures referenced in the analyses below were never planned to be assessed at 6-month follow-up, consistent with the Method section and Figures of the original publication.

**Table 1:** SPIRIT Depiction of Timing and Measures Collected for Implementation Phase

	**Screening**	**Post-Training**	**Pre-Treatment**	**Mid-Treatment**	**Post-Treatment**	**6-Months Post-Treatment**
**Patient**						
Sociodemographics			x		x	x
Eligibility Items	x					
PROMIS-SD^P^	x		x	x	x	x
PROMIS-SRI			x	x	x	x
DSM-5 Cross-Cutting			x		x	x
SDS			x		x	x
Sleep Health Composite			x	x	x	x
PHENX Toolkit			x		x	x
CEQ					x	
**Provider**						
Sociodemographics		x				
Occupation		x				
Acceptability^P^		x		x	x	
Appropriateness		x		x	x	
Feasibility		x		x	x	
Number of Sessions					x	

**Note:** Allocation to Adapted or Standard TranS-C occurs at the county level and prior to enrollment of any participants in that county (i.e., patients or providers). Enrollment of patients and allocation to immediate TranS-C or delayed TranS-C (UC-DT) occur after the screening and before the pre-treatment assessment. Enrollment of providers after the training

**Note:** PROMIS-SD is only assessed during the pre-treatment assessment if done more than one month after the screening to minimize burden for patients

^P^ Primary Outcome, *PROMIS-SD* PROMIS-Sleep Disturbance, *PROMIS-SRI* PROMIS-Sleep Related Impairment, *SDS* Sheehan Disability Scale, *CEQ* Credibility/Expectancy Questionnaire

The incorrect and correct information is shown in this correction article. The original article has been updated [1].

**Incorrect**

- The level 1 equation will include dummy-coded time indicators as the predictor (0 = pre-treatment, 1 = post-treatment, and 2 = 6FU)…Significant interactions will be interpreted using planned contrasts (i.e., treatment effects on change from pre-treatment to post-treatment and pre-treatment to 6FU) and graphs.
- Linear regression will be used to test treatment condition (Adapted vs. Standard TranS-C) predicting patient perceptions of TranS-C’s credibility at post-treatment and 6FU.
- The level 1 equation will include the moderator and dummy-coded time indicators as the predictors (0 = pre-treatment, 1 = post-treatment, and 2 = 6FU). A significant interaction indicates a moderating effect and will be probed with planned contrasts (e.g., moderating effects on the differences between treatments in change from pre-treatment to post-treatment or pre-treatment to 6FU) and graphs.

**Correct**

- The level 1 equation will include dummy-coded time indicators as the predictor (0 = post-training, 1 = post-treatment)…Significant interactions will be interpreted using planned contrasts (i.e., treatment effects on change from post-training to post-treatment) and graphs.
- Linear regression will be used to test treatment condition (Adapted vs. Standard TranS-C) predicting patient perceptions of TranS-C’s credibility and perceived improvement at post-treatment.
- The level 1 equation will include the moderator and dummy-coded time indicators as the predictors (0 = pre-treatment, 1 = post-treatment). A significant interaction indicates a moderating effect and will be probed with planned contrasts (e.g., moderating effects on the differences between treatments in change from pre-treatment to post-treatment) and graphs.
